# Efficacy of Cold Plasma for Direct Deposition of Antibiotics as a Novel Approach for Localized Delivery and Retention of Effect

**DOI:** 10.3389/fcimb.2019.00428

**Published:** 2019-12-20

**Authors:** Agata Los, Dana Ziuzina, Daniela Boehm, Lu Han, Denis O'Sullivan, Liam O'Neill, Paula Bourke

**Affiliations:** ^1^Plasma Research Group, School of Food Science and Environmental Health, Technological University Dublin, Dublin, Ireland; ^2^TheraDep, Questum Centre, Clonmel, Ireland; ^3^School of Biological Sciences, Queens University Belfast, Belfast, United Kingdom

**Keywords:** cold plasma, antibiotics, *E. coli*, *P. aeruginosa*, biofilm, localized delivery

## Abstract

Antimicrobial coating of medical devices has emerged as a potentially effective tool to prevent or ameliorate device-related infections. In this study the plasma deposition process for direct deposition of pharmaceutical drugs on to a range of surfaces and the retention of structure function relationship and antimicrobial efficacy against mono-species biofilms were investigated. Two selected sample antibiotics—ampicillin and gentamicin, were deposited onto two types of surfaces—polystyrene microtiter plates and stainless steel coupons. The antimicrobial efficacy of the antibiotic-coated surfaces was tested against challenge populations of both planktonic and sessile *Escherichia coli* and *Pseudomonas aeruginosa*, with responses monitored for up to 14 days. The plasma deposition process bonded the antibiotic to the surfaces, with localized retention of antibiotic activity. The antibiotics deposited on the test surfaces retained a good efficacy against planktonic cells, and importantly prevented biofilm formation of attached cells for up to 96 h. The antibiotic rapidly eluted from the surface of antibiotic-coated surfaces to the surrounding medium, with retention of effect in this surrounding milieu for up to 2 weeks. Control experiments established that there was no independent antimicrobial or growth promoting effect of the plasma deposition process, where there was no antibiotic in the helium plasma assisted delivery stream. Apart from the flexibility offered through deposition on material surfaces, there was no additive or destructive effect associated with the helium assisted plasma deposition process on the antibiotic. The plasma assisted process was a viable mean of coating clinically relevant materials and developing innovative functional materials with retention of antibiotic activity, without employing a linker or plasma modified polymer, thus minimizing bio-compatibility issues for medical device materials. This offers potential to prevent or control instrumented or non-permanent device associated infection localized to the surgical or implant site.

## Introduction

Medical device-associated infections caused by antibiotic-resistant pathogens are a global public health concern driving research toward new antibiotics, antibiotic alternatives, and mechanisms to reinstate antibiotic activity. Such infections are generally caused by microorganisms that form biofilms on various devices including orthopedic implants, central venous catheters, and urinary catheters (Buhmann et al., 2016). The microorganisms frequently associated with medical-device related infections include both Gram-positive bacteria, such as *Staphylococcus aureus, Staphylococcus epidermidis*, and *Enterococcus faecalis*, and Gram-negative bacteria (Percival et al., 2015)—most frequently *Escherichia coli* and *Pseudomonas aeruginosa*. *E. coli* was one of the first causes of orthopedic implant infections (Crémet et al., 2015), whereas the opportunistic pathogen *P. aeruginosa* is a frequent gram-negative etiologic agent associated with infections of indwelling catheters and foreign body implants (Brouqui et al., 1995). *Pseudomonas aeruginosa* is often associated with chronic biofilm infections and it has become a model organism for biofilm research (Harmsen et al., 2010; Christensen et al., 2013).

The consequences of implant-associated infections are significant and in most cases require revision surgery, with removal of the implant and prolonged antibiotic treatment (Ketonis et al., 2012). Molecular antibiotics traditionally used against bacteria face several disadvantages, including the worldwide emergence of antibiotic resistance, difficulty of incorporation into many materials and sensitivity to harsh environments encountered during industrial processes (Levy and Marshall, 2004), therefore, new approaches of drug delivery are under investigation. The main methods to decrease incidence of device-related infections include anti-adhesive biomaterials using physicochemical surface modification methods, e.g., non-drug containing coatings, films and ion treatments, or, direct incorporation of active compound into or onto the medical device (Wu and Grainger, 2006). The purpose of these bioactive surfaces is to disrupt the metabolic activity of the microbes or to prevent bacterial adhesion to the implant and, consequently, to inhibit the development of biofilm (Ketonis et al., 2012). Wu and Grainger (2006) list benefits of local drug release strategies over systemic drug therapy as: lower doses of antimicrobial agents needed for treatment, extended period of controlled release from surfaces of devices directly to site, lack of systemic drug exposure, as well as less likelihood of promoting antibiotic resistance.

An emerging approach for drug delivery is cold plasma technology, which is widely employed for materials surface modifications. In contrast to “wet” chemistry methods which depend strongly on substrate properties, plasma deposition of antimicrobial coatings can be applied to a broad range of polymers, ceramics, and metal surfaces (Nikiforov et al., 2016) and the coating may be adhered to the surface without the use of linkers, binders, or polymers that may impact biocompatibility. In this study, the effect of a cold plasma deposition process on antibiotics was determined, examining the impact on the antibiotic functional properties, specifically the retention of antimicrobial activity, the retention of efficacy, comparison with controls, and evaluation of elution from the antibiotic coated surface. Ampicillin and gentamicin were selected and deposited onto two types of surfaces—polystyrene microtiter plates and stainless steel coupons. Although safety of local administration of gentamicin has been debated and tested in recent studies, this antibiotic was incorporated in the present study as it has been widely used in medicinal applications over the past 50 years and due to its wide spectrum of bactericidal activity against both gram-negative and gram-positive organisms, including staphylococci (Chen et al., 2014). The efficacy of antibiotic-coated surfaces was tested against both *E. coli* and *P. aeruginosa*.

## Materials and Methods

### Bacterial Strains and Inocula Preparation

*Escherichia coli* ATCC 25922 and *P. aeruginosa* ATCC 27853 were obtained from the microbiology stock culture of the School of Food Science and Environmental Health of the Dublin Institute of Technology. Stock cultures in the form of protective beads were maintained at −70°C and working inoculum was prepared as described previously (Los et al., 2017). Briefly, one protective bead of culture of *E. coli* and *P. aeruginosa* was streaked onto separate tryptic soy agar (TSA, Biokar Diagnostics, France) plate and incubated at 37°C for 24 h. A single isolated colony of either *E. coli* or *P. aeruginosa* was inoculated into Mueller Hinton Broth (MHB, Oxoid LTD, UK) and incubated overnight at 37°C. Suspensions were washed twice in phosphate-buffered saline (PBS, Sigma-Aldrich Co., Ireland) and bacterial suspensions (6–7 log CFU/ml) in either MHB or PBS were prepared using 0.5 McFarland standard (BioMerieux, Marcy-l'Etoile, France). Inoculum concentration was confirmed by plating appropriate dilutions on TSA and incubation at 37°C for 24 h.

### Biofilm Formation

The effect of deposited antibiotic on 24 h bacterial monoculture biofilms was investigated. Biofilms were formed by adding 200 μl of prepared bacterial suspension (7 log CFU/ml) of either *E. coli* or *P. aeruginosa* into the wells of a 96-well flat bottom microtiter plate (Sarstedt, Nümbrecht, Germany). Plates were incubated at 37°C for 24 h and after that the supernatant containing suspended planktonic cells was removed. Plates were washed gently twice with PBS to remove any planktonic cells (Los et al., 2017).

### Preparation of Stock and Working Solutions of Antibiotics

Stock solutions of ampicillin and gentamicin (Sigma-Aldrich, Ireland) were prepared using sterile deionized water and further stored at 4°C. Powder antibiotics were stored in the temperature indicated by the drug manufacturer. For antibiotic susceptibility tests of planktonic cells and bacterial biofilms, stock solutions were diluted in MHB to obtain working solutions two times more concentrated than the final highest drug concentration used in the experiment (Table 1). For plasma deposition on microtiter plates and steel coupons, antibiotic solutions (concentration −50 mg/ml) were prepared using sterile deionized water.

**Table 1:** Concentration ranges of antibiotics used in the study.

**Antibiotic**	**MIC (μg/ml)**		**Biofilm testing (μg/ml)**	
	***E. coli***	***P. aeruginosa***	***E. coli***	***P. aeruginosa***
Ampicillin	0.25–128	NA	8–256	NA
Gentamicin	0.031–16	0.031–16	2–64	1–32

*NA, not applicable*.

### Antibiotic Susceptibility of Planktonic Cells and Biofilms

Minimum inhibitory concentration (MIC) and the effects on biofilm formation in the presence of antibiotics were determined. Concentration ranges used for each antibiotic and microorganism are listed in Table 1. For determination of MIC, broth micro-dilution method for antibacterial testing was used as recommended by the Clinical and Laboratory Standards Institute protocol (CLSI, 2012). The assay was performed according to the protocol of Sarker et al. (2007) with minor modifications. A concentration gradient of antibiotic was created in 96-well flat bottom microtiter plates (Sarstedt, Nümbrecht, Germany) −100 μl of antibiotic in MHB in concentration two times higher than the highest concentration used in the experiment was added into the wells in the first row, while 50 μl of MHB with no antibiotic was added into the remaining wells. Serial dilutions were performed by pipetting 50 μl of antibiotic solution from row to row in decreasing concentrations. Finally, 50 μl of bacterial suspension (6 log CFU/ml) of either *E. coli* or *P. aeruginosa* were inoculated into each well to achieve a final cell concentration of 5 × 10^5^ CFU/ml. Additionally, plates included a negative control (MHB only) and a positive bacterial growth control. Plates were incubated for 24 h at 37°C. Bacterial growth curves in the presence of antibiotic were determined spectrophotometrically using optical density at 600 nm. The MIC value was defined as the lowest antibiotic concentration that caused no bacterial growth.

To determine the effect of antibiotic concentration range on biofilm formation, antibiotic solutions (Table 1) diluted in MHB were added into the wells containing preformed biofilms. Plates were further incubated at 37°C for 72 h (total incubation time −96 h). The supernatant was removed, plates were washed gently twice with PBS and biofilms air-dried for further analysis.

### Effect of Antibiotic-Coated Surfaces on Microbial Growth

#### Plasma Deposition of Antibiotics on Microtiter Plates and Steel Coupons

**Figure 1:**
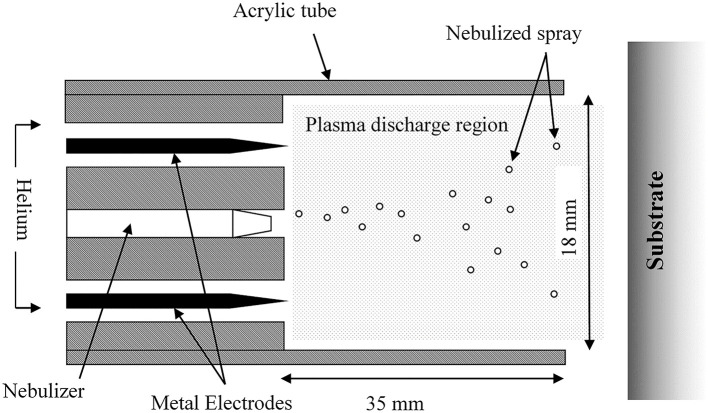
Plasma deposition system.

**Figure 2:**
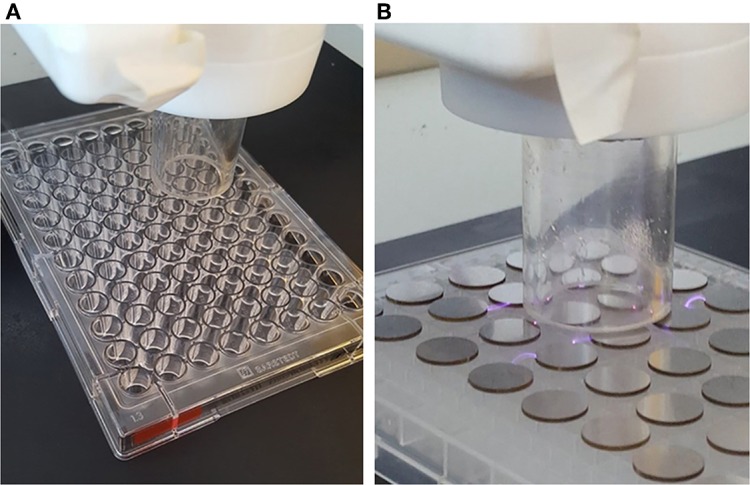
Plasma assisted deposition on **(A)** 96-well microtiter plates, **(B)** steel coupons.

Plasma deposition was carried out using a purpose build deposition system comprising a Redline G2000 High Voltage power supply connected to a custom build Teflon deposition unit encasing two metal electrodes. a pneumatic nebulizer (T2100, Burgener Research) was placed between the electrodes and this was connected to a syringe pump to provide a constant flow of gentamicin sulfate aqueous solution (50 mg/ml). The liquid was nebulized using a helium flow of ~3 slm. In addition, a separate helium flow of 8 slm was provided to the metal electrodes to create the plasma discharge. The plasma discharge was combined with the nebulized droplet spray in an acrylic tube (18 mm inner diameter × 35 mm length) and the substrates to be coated were placed at the outlet of the tube. This is shown schematically in Figure 1. For larger areas or multiple smaller coupons, the plasma was mounted on a computer numerically controlled table and moved in a raster pattern over the target surface as shown in Figure 2. Different antibiotic concentrations on the surface were obtained by coating the plates with 1, 3, and 5 layers of each antibiotic. Steel coupons were coated with 18 layers of gentamicin (9 layers on each side of the coupon). After plasma-deposition, both microtiter plates and steel coupons were stored at 4°C.

#### Bacterial Growth and Biofilm Formation on Antibiotic-Coated Microtiter Plates

Two hundred microliter of prepared bacterial suspension (7 log CFU/ml) of either *E. coli* or *P. aeruginosa* in MHB were added into the wells of plasma-treated plates with no active component and antibiotic plasma-coated plates to consider if the plasma coating process had any additional antimicrobial or growth promoting effect, bacterial growth on untreated plates was also tested. Plates were incubated at 37°C for 24 h and the growth of planktonic cells was monitored via optical density measurements. In a separate set of experiments, plates were incubated at the same temperature for 96 h—after this time, biofilm formation on the plates was monitored using crystal violet, XTT and plate count assays.

#### Antimicrobial Efficacy of Gentamicin-Coated Steel Coupons

Blank plasma-treated steel coupons (control) or gentamicin plasma-coated coupons (ACP-G) were transferred into separate sterile tubes containing 10 ml of bacterial suspension (7 log CFU/ml) in either PBS or MHB. Suspensions were further incubated up to 24 h at 37°C. Surviving populations of cells were assessed at 0, 1, 2, 4, 6, and 24 h of incubation by withdrawing 0.1 ml and 1 ml of cell suspension from the tube followed by plating appropriate dilutions on TSA. Growth of planktonic cells in the presence of plasma-treated and gentamicin-plasma coated steel coupons was compared to controls with no coupons.

#### Elution Tests and Stability of Gentamicin-Coated Steel Coupons During dry Storage

The detachment of gentamicin from steel coupons, elution into liquid supernatant and antibiotic stability was assessed over a period of 14 days. Coupons were submerged in tubes containing 10 ml of either a buffer solution (PBS) or nutrient rich medium (MHB) (1 coupon per tube) and incubated at 37°C up to 14 days. At each of 7 time points (days: 0, 1, 2, 4, 7, 10, and 14), two coupons from two randomly selected separate tubes were removed and antimicrobial efficacy of the solutions against planktonic cells and biofilms was tested. The final volume of antibiotic eluate in both PBS and MHB solutions was 37.5% (v/v) of the total test volume (Table 2).

**Table 2:** Tests with solutions containing gentamicin released from the plasma deposited steel coupon into PBS and MHB.

**Suspension**	**Planktonic cells**		**Biofilm testing**	
	**Coupons in PBS**	**Coupons in MHB**	**Coupons in PBS**	**Coupons in MHB**
Bacterial suspension in PBS	50 μl	50 μl	–	–
Antibiotic solution	75 μl (PBS-G)	75 μl (MHB-G)	75 μl (PBS-G)	75 μl (MHB-G)
Other	75 μl MHB	75 μl PBS	75 μl MHB	125 μl PBS
			50 μl PBS	
Total volume in each well	200 μl	200 μl	200 μl	200 μl

*PBS-G, solution containing gentamicin released from the steel coupon into PBS; MHB-G, MHB solution containing gentamicin released from the steel coupon into MHB*.

To quantify the effect of these antibiotic solutions in either PBS or MHB on planktonic cells, 2-fold dilutions were prepared on the plates as previously described MIC procedure. Serial dilutions were performed by pipetting 75 μl of antibiotic solution from row to row in serially decreasing concentrations (volume of antibiotic suspension in first row −150 μl). To obtain the same amount of nutrient rich medium (MHB) in each well, solutions containing gentamicin released from the steel coupons into PBS were filled with 75 μl of MHB, whereas gentamicin-containing MHB solutions were supplemented with 75 μl of PBS (Table 2). Finally, 50 μl of bacterial suspensions in PBS (2 × 10^6^ CFU/ml) of either *E. coli* or *P. aeruginosa* were inoculated into each well to achieve a final cell concentration of 5 × 10^5^ CFU/ml. Plates were incubated at 37°C for 24 h and the growth of planktonic cells was monitored via optical density measurements. Additionally, to provide an insight into the stability of antibiotic coating, antimicrobial efficacy of the solutions containing antibiotic released from the coupons against planktonic cells was investigated for coupons stored for 3 weeks at 4°C (assessed immediately after placing coupon into the liquid—day 0).

The effect of the released antibiotic solutions in either PBS or MHB on bacterial biofilms was also quantified. Twenty hours biofilms were prepared as described in section Antibiotic susceptibility of planktonic cells and biofilms. Antibiotic solutions were added into the wells containing biofilms. Subsequently, the wells were filled with either PBS or MHB to obtain the same amount of MHB in each well (Table 1). Plates were further incubated at 37°C for 72 h (total incubation time −96 h). Finally, the supernatant was removed, plates were washed gently twice with PBS and biofilms air-dried were further analyzed.

#### Calculation of Gentamicin Deposition Concentration

The starting solution was 50 mg/ml gentamicin in sterile water. The flow rate for the deposition was 45 ul/min, resulting in 2.25 mg entering the system per minute (0.045 ml/min ^*^ 50 mg/ml). The coating time for the platform has been established as 245 s. The platform size was 0.085 m ^*^ 0.125 m = 106.25 cm^2^. Using the time required to coat the platform and the concentration entering the system at a defined flow rate, the concentration per layer was 9.18 mg. Taking 9.18 mg per 106.25 cm2, this yields 0.0864 mg/cm2. The system efficiency has been previously calculated at 30–40%, thus yielding a range of 0.02592–0.03456 mg/cm2. For a 10 mm coupon; π *r*^2^ = π 0.005 m^2^ = 0.7854 cm2. At 30% efficiency: 0.7854 cm2 ^*^ 0.02592 mg = 0.0204 mg per coupon, per layer, and at 40% efficiency: 0.7854 cm2 ^*^ 0.03456 mg = 0.0271 mg per coupon, per layer. Applying nine layers per coupon side yielded 0.3672–0.4878 mg of gentamicin per coupon.

### Microbiological Analysis

#### Optical Density Measurements

Growth of planktonic cells and bacterial survivability in presence of antibiotics was monitored by optical density (600 nm) measurements using a micro-plate reader (Synergy HT, Biotek Instruments Inc.) during 24 h of incubation.

#### Crystal Violet Assay

The crystal violet (CV) assay was used for the quantification of bacterial biomass and was performed according to the procedure described in Peeters et al. (2008) with minor modifications. For fixation of the biofilms, 100 μl of 99% methanol was added (15 min), after which supernatants were removed and the plates were air-dried. Then, 100 μl of a 0.2% CV solution (Merck, Portugal) was added to all wells. After 20 min, the excess free CV was removed by rinsing the plates. Finally, bound CV was released by adding 150 μl of 33% acetic acid (Sigma Aldrich, Ireland). The absorbance was measured at 590 nm using a microplate reader. All steps were carried out at room temperature. Each absorbance value was corrected by subtracting the means of absorbance of a blank (uninoculated medium).

#### XTT Assay

The XTT assay was used to quantify metabolic activity and was carried out according to the procedure described in Peeters et al. (2008). Before each assay, fresh solutions of 2,3-bis(2-methoxy-4-nitro-5-sulfophenyl)-5-[(phenylamino)carbonyl]-2H-tetrazolium hydroxide (XTT, 1 mg/ml, Sigma-Aldrich Co., Ireland) were prepared by dissolving 4 mg in 10 ml of pre-warmed PBS. The solution was supplemented with 5.5 mg menadione (Sigma-Aldrich Co., Ireland) in 10 ml acetone (Sigma-Aldrich Co., Ireland). The wells containing biofilms and negative controls (media without inocula) were filled with sterile PBS (100 μl) and then XTT-menadione (100 μl) was added. Plates were incubated for 5 h at 37°C in the dark. After incubation, the supernatant (100 μl) from each well was transferred into the wells of a new 96-well microtiter plate and the absorbance at 486 nm was measured. Each absorbance value was corrected by subtracting the means of absorbance of a blank (uninoculated medium).

### Contact Angle Measurements

To investigate the changes of surface characteristics of steel coupons after plasma deposition, the apparent contact angles of deionized water (Sigma Aldrich, Ireland) on the steel samples were measured by the sessile drop technique using a contact angle meter (Theta Lite Optical Tensiometer, Biolin Scientific, UK). Analysis was performed immediately after deposition of a single droplet on the coupon surface. The images were recorded at 15 frames per second for 10 s and analyzed using the OneAttension software (v 2.1). All the values reported are the mean of more than 100 data points done in triplicates. The contact angle measurements from within the wells of the 96 well microplates were conducted using a Kruss TVA100 top view analyzer. The analysis used a 0.5 μl drop of deionized water and measurements were recorded 60 s after placing the drop in the well. All analysis was conducted with the Kruss ADVANCE software package.

### Fourier Transform Infra-Red (FTIR) Spectroscopy

FTIR spectroscopy was carried out by applying the gentamicin sulfate coatings directly onto NaCl discs. For the non-plasma deposited sample, the solution was sprayed at a flow rate of 45 μL/min for 60 s and then allowed to dry in air. Plasma deposition was carried out as described in section Plasma deposition of antibiotics on microtiter plates and steel coupons using NaCl discs as the substrate with a static deposition time of 60 s. FTIR spectra were then collected on a Perkin Elmer Spectrum 2000 instrument operating in single beam mode using 64 scans and a 0.5 cm^−1^ resolution.

### Statistical Analysis

Statistical analysis was performed by analysis of variance (ANOVA) using IBM SPSS statistics 25 Software (SPSS Inc., Chicago, USA) according to the method of Fisher's Least Significant Difference-LSD at the 0.05 level.

## Results

### Sensitivity of *E. coli* and *P. aeruginosa* to Selected Antibiotics—Planktonic Susceptibility Testing and the Effect of Antibiotics on Biofilm Formation

The susceptibility of *E. coli* and *P. aeruginosa* to selected antibiotics was confirmed to benchmark the antibiotic effects. The MIC values are presented in Table 3. The interpretation of the MIC values was based on the Clinical and Laboratory Standards Institute (CLSI) guidelines published in annual supplement Performance Standards for Antimicrobial Susceptibility Testing (M100-S28) (CLSIC and LSI, 2018).

**Table 3:** The MIC values of selected antibiotics tested against *E. coli* and *P. aeruginosa*.

**Antibiotic**	***E. coli***		***P. aeruginosa***	
	**MIC (μg/ml)**	**Interpretation**	**MIC (μg/ml)**	**Interpretation**
Ampicillin	8	S	NA	NA
Gentamicin	2	S	4	S

*S, susceptible; NA, not applicable*.

**Figure 3:**
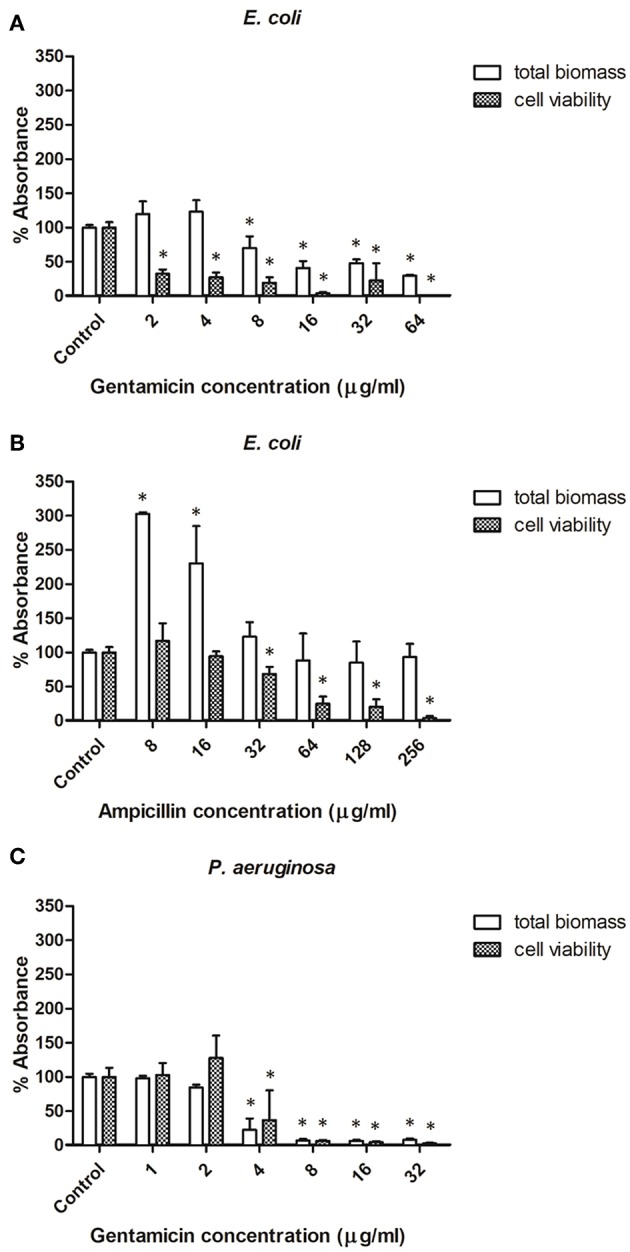
Effect of antibiotic concentration on bacterial biofilm formation. **(A,B)** Biofilms formed by *E. coli* in presence of ampicillin and gentamicin, respectively. **(C)** Biofilms formed by *Pseudomonas aeruginosa* in presence of gentamicin. ^*^ indicates a significant difference between the corresponding untreated control and samples treated with antibiotics (*p* < 0.05).

The effect of selected antibiotics against 24-h-old biofilms is shown in Figure 3, with total biofilm biomass and metabolic activity assessed by crystal violet (CV) and XTT assays, respectively. In general, biofilm challenges are less susceptible to antibiotics than planktonic cells of bacteria (Hall and Mah, 2017). In the case of *E. coli* biofilms exposed to gentamicin, a concentration of 8 μg/ml and above significantly decreased biomass production, whereas all tested concentrations (2–64 μg/ml) negatively affected bacterial metabolic activity (Figure 3A). Lower concentrations of ampicillin (8 and 16 μg/ml) enhanced biomass production of *E. coli*, whereas concentrations of 32 μg/ml and above caused a significant decrease of metabolic activity within *E. coli* biofilms, but did not affect biomass (Figure 3B). This phenomenon is not unique to ampicillin - it has been widely reported in literature that sublethal concentrations of antibiotics can cause metabolic and physiological changes indicating that the organism is preparing to withstand lethal antibiotic concentrations, which can lead to e.g., stimulation of biofilm formation at lower doses of antibiotics (Nguyen et al., 2014; Knudsen et al., 2016). For *P. aeruginosa* biofilms treated with gentamicin, similar trends were observed for both CV and XTT assays—a concentration of 4 μg/ml and above significantly decreased biomass production and metabolic activity (Figure 3C).

### Effect of Plasma Coating Process With No Active Component

To determine if plasma coating itself had any antimicrobial or growth promotion effect, plasma treatment was applied to two types of surfaces—microtiter plates and steel coupons, with no active component included in the nebulizer stream. No significant differences were recorded between the growth on untreated or plasma-treated microtiter plates, monitored via optical density measurements over 24 h. Similarly, the planktonic growth of *E. coli* and *P. aeruginosa* was not significantly affected in the presence of plasma-treated coupons as compared to controls with no coupons (data not shown).

### Effect of Plasma Coating Surfaces With Antibiotic on Microbial Growth

#### Microtiter Plates

The next stage of analysis was performed using microtiter plates coated with ampicillin and gentamicin. The effect of plasma deposited antibiotics on planktonic growth and subsequently on biofilm formation and viability of the two challenge microorganisms—*E. coli* and *P. aeruginosa*, was investigated. Growth of *E. coli* was tested using plates coated with either ampicillin or gentamicin, whereas growth of *P. aeruginosa*—with gentamicin only. Multiple deposition layers of antibiotics (1, 3, and 5) on the plates were compared.

**Figure 4:**
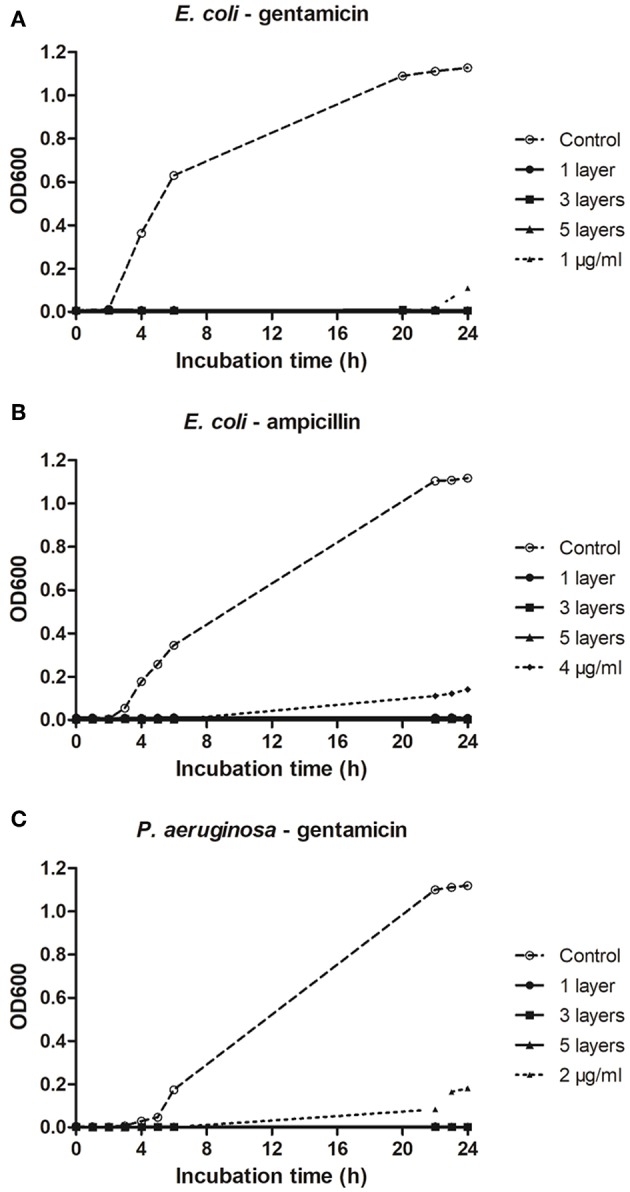
Effect of plasma deposited antibiotics on micro-titer plates on growth of planktonic *E. coli* and *P. aeruginosa*. **(A,B)** Growth of *E. coli* on microtiter plates deposited with ampicillin and gentamicin, respectively. **(C)** Growth of *P. aeruginosa* on microtiter plates deposited with gentamicin.

No growth of planktonic cells of *E. coli* was recorded after 24 h of incubation in plates coated with either ampicillin (Figure 4A) or gentamicin (Figure 4B). Similarly, gentamicin deposited onto plates completely inhibited growth of *P. aeruginosa* (Figure 4C). In all cases, one layer of an antibiotic was sufficient to prevent bacterial growth. As quantifying the exact amount of antibiotic deposited on a microtiter plate was not possible, antimicrobial efficacy of the antibiotics deposited on the plates were compared to calibration curves to provide an indication of the concentration range of functional antibiotic deposited during the process.

**Figure 5:**
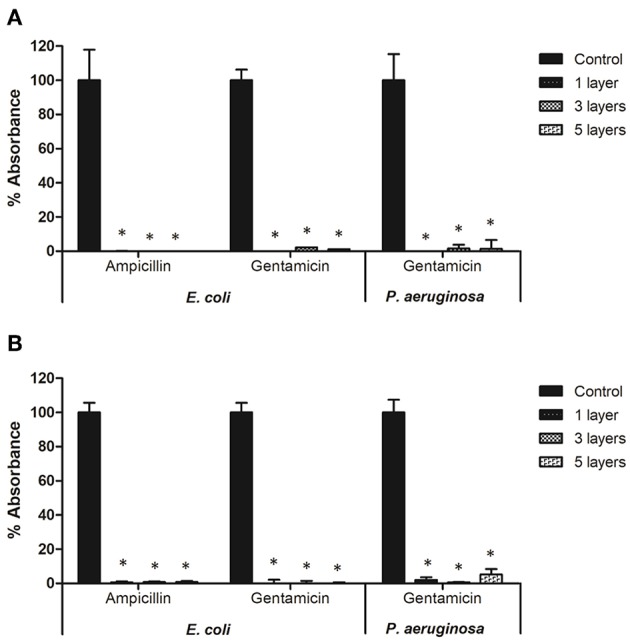
Biofilm formation of *E. coli* and *P. aeruginosa* on microtiter plates coated with antibiotics: **(A)** total biomass and **(B)** cell viability. ^*^ indicates a significant difference between the corresponding untreated control and biofilms formed on microtiter plates coated with antibiotics (*p* < 0.05).

Plasma deposited antibiotics significantly inhibited bacterial biofilm formation on microtiter plates (Figure 5). On plates coated with ampicillin no biomass production was recorded for *E. coli*, and metabolic activity was lower than 2% of that observed for controls. Regardless of the number of layers of antibiotic deposited, there was <0.5 and 0.8% of biomass production and metabolic activity, respectively, for *E. coli* biofilms grown on gentamicin coated plates. Using gentamicin-plasma coated plates decreased *P. aeruginosa* metabolic activity to <6% for all antibiotic layers tested, and completely inhibited biomass production.

#### Bacterial Growth in the Presence of Gentamicin-Plasma Coated Coupons

Plasma-deposited gentamicin on steel coupons was effective at reducing the populations of planktonic cells of bacteria—the antibiotic efficacy was retained after the plasma deposition process and the antimicrobial effect was shortly apparent in the surrounding liquid milieu of PBS or MHB.

**Figure 6:**
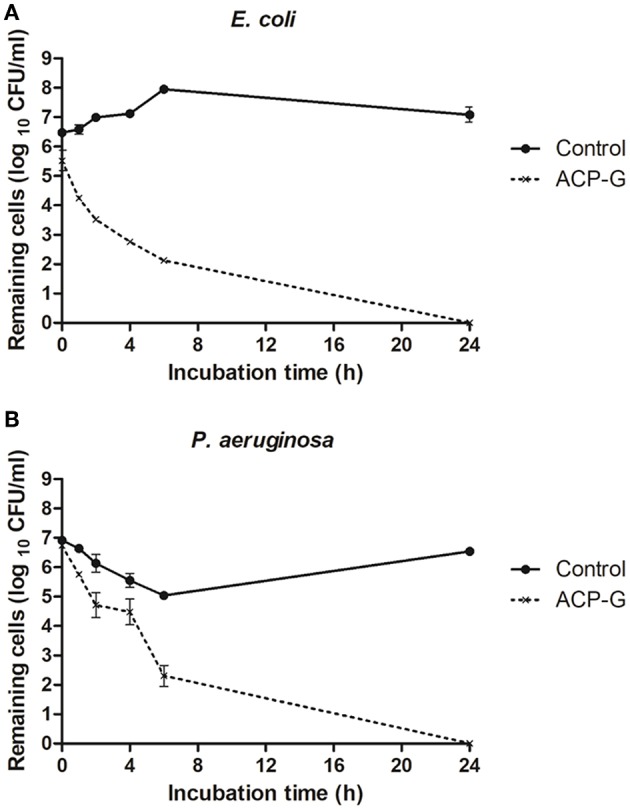
Growth of planktonic cells of: **(A)** *E. coli* and **(B)** *P. aeruginosa* in presence of plasma-treated steel coupons (control) and steel coupons plasma-coated with gentamicin (ACP-G) submerged in PBS.

**Figure 7:**
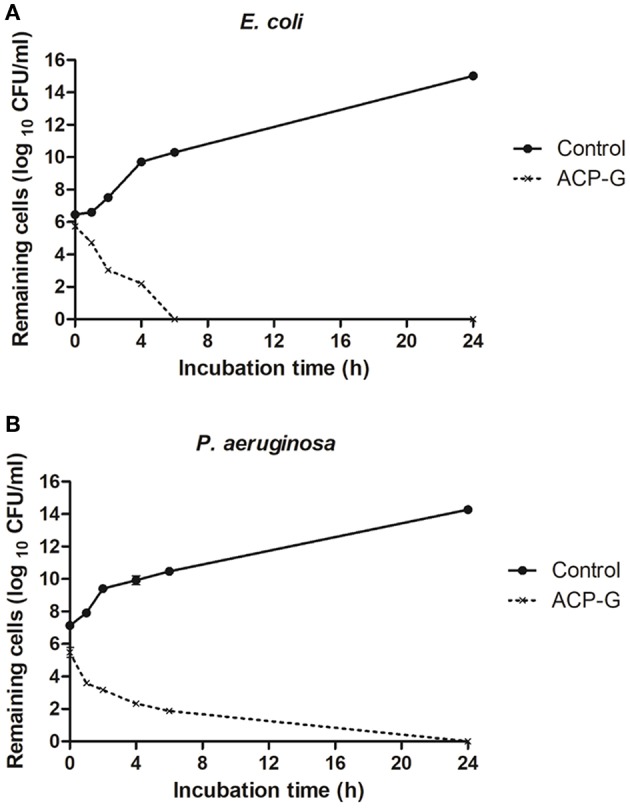
Growth of planktonic cells of: **(A)** *E. coli* and **(B)** *P. aeruginosa* in presence of plasma-treated steel coupons (Control) and steel coupons plasma coated with gentamicin (ACP-G) submerged in MHB.

Submerging gentamicin-coated coupons in PBS with a defined bacterial load had an immediate antimicrobial effect. Populations of *E. coli* and *P. aeruginosa* were reduced by 1.0 and 0.2 log_10_ CFU/ml (Figure 6), respectively in PBS, and by 0.7 and 1.7 log_10_ CFU/ml (Figure 7) in MHB. Within 4 h, 3.7 and 2.4 log_10_ CFU/ml reductions were achieved for *E. coli* and *P. aeruginosa*, respectively in PBS. Cells of both strains were undetectable from the media by 24 h (Figure 6). Antimicrobial efficacy of gentamicin-coated coupons was more pronounced in MHB. Within 4 h, 4.3 and 4.8 log_10_ CFU/ml reductions were achieved for *E. coli* and *P. aeruginosa*, respectively. Complete inactivation was achieved for *E. coli* and *P. aeruginosa* within 6 and 24 h, respectively (Figure 7).

### Elution of Gentamicin From Steel Coupons

Antibiotic detachment and elution into the surrounding liquid was assessed to inform the stability and activity of plasma coatings. Gentamicin-deposited coupons were submerged in either PBS or MHB and the antimicrobial activity of aliquots of the resulting solutions against both planktonic cells and bacterial biofilms were tested over a period of 14 days.

**Figure 8:**
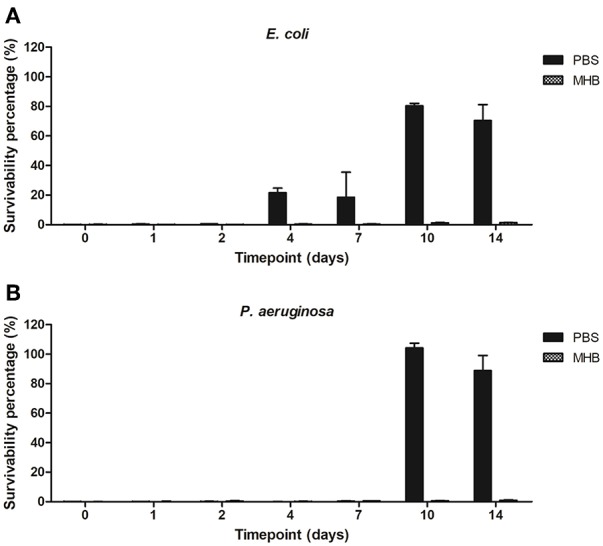
Survivability percentage of planktonic cells of **(A)** *E. coli* and **(B)** *P. aeruginosa* in presence of solutions containing gentamicin released from the plasma coated steel coupons.

Antimicrobial activity of PBS-gentamicin solution against planktonic cells of *E. coli* remained unaffected for 2 days (<1% survivability) with a potential to be used for infection control up to 7 days (<25% survivability). PBS-gentamicin solution incubated for 10 and 14 days resulted in a decrease of the number of planktonic cells of *E. coli* by 20 and 30%, respectively. Efficacy of PBS-gentamicin solution tested against *P. aeruginosa* was stable for up to 7 days with <0.5% survival recorded. The MHB-gentamicin solutions showed higher antimicrobial activity than PBS-gentamicin solutions—with <0.5% cell survivability for *E. coli* achieved up to 7 days, and <2% survival for solutions incubated for both 10 and 14 days. Similarly, efficacy against *P. aeruginosa* was retained for up to 14 days (<1.5 % survivability). Overall, MHB-gentamicin solution showed high activity both against *E. coli* and *P. aeruginosa* for 14 days (Figure 8).

**Figure 9:**
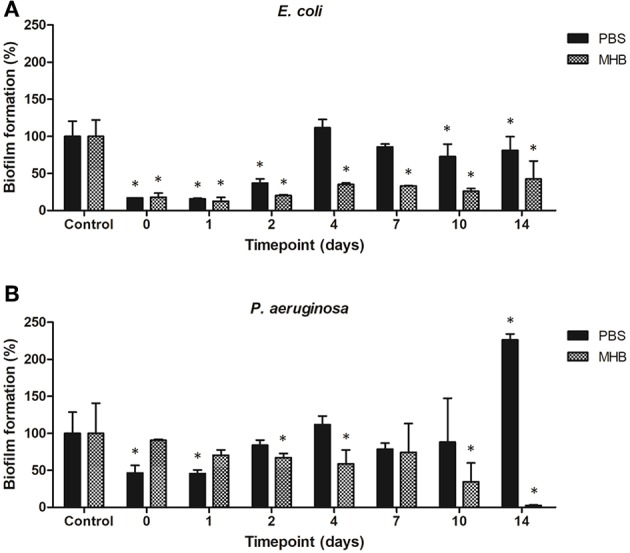
Biofilm formation of **(A)** *E. coli* and **(B)** *P. aeruginosa* in presence of solutions containing gentamicin eluted from the steel coupons over 14 days. ^*^indicates a significant difference between the corresponding untreated control and biofilms formed in presence of solutions containing antibiotics (*p* < 0.05).

The potential for biofilm formation in the presence of solutions containing gentamicin eluted from the steel coupons into PBS and MHB was investigated. PBS solutions containing eluted gentamicin inhibited biomass production when incubated up to 2 days for *E. coli* (<40% total biomass as compared to control biofilms) and 1 day for *P. aeruginosa* (<50% total biomass). Using PBS-gentamicin solution eluted for 14 days promoted biomass production of *P. aeruginosa*. MHB-gentamicin solutions inhibited biomass production of *E. coli* by 80% up to 2 days and by 55–75% up to 14 days. MHB –gentamicin eluted for 14 days inhibited 97% of *P. aeruginosa* biomass production (Figure 9).

### Stability of Gentamicin on Steel Coupons During Dry Storage

A further interrogation of this process was to examine the stability of plasma coated antibiotics on surfaces over time; gentamicin deposited on steel coupons remained stable up to 3 weeks post plasma deposition, i.e., no growth after 24 h of incubation was observed for planktonic cells of *E. coli* and *P. aeruginosa* in the presence of gentamicin released into PBS and MHB from the steel coupons stored for 3 weeks at 4°C (data not shown). Solutions were tested immediately upon submerging a coupon into a liquid.

### Changes of Surface Substrate Characteristics After Plasma Deposition

It was observed that the surface of steel coupons subjected to plasma deposition of antibiotics increased its hydrophilicity which can be observed by a decrease of water contact angle. After plasma deposition, water contact angle (WCA) was decreased by ~20% (Table 4). A reduction in the WCA measurement was also detected on the coated microplate samples, though the decrease was significantly less pronounced than on the steel. This may reflect the lower initial contact angle of the tissue culture microplate (Table 5).

**Table 4:** Effect of plasma deposition on water contact angle (WCA) and surface free energy (γtot) values of steel coupons surface.

**Sample**	**WCA (°)**	**γtot [mN/m]**	**Image**
Control steel coupon	96.4^a^ ± 2.0	25.3^a^ ± 1.2	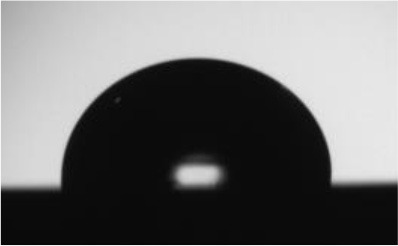
Plasma-coated	75.8^b^ ± 3.6	38.1^b^ ± 2.2	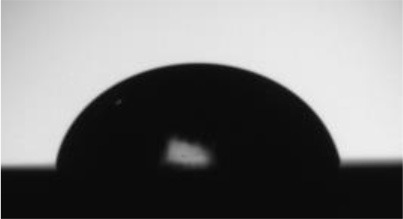

*Different letters indicate significant difference between the control and plasma-coated coupons (p <0.05)*.

**Table 5:** Effect of plasma deposition on water contact angle (WCA) values of polystyrene microplate surfaces.

**Sample**	**WCA (°)**
Control surface	58.3 ± 2.2
1 layer of plasma coating	57.2 ± 2.5
3 layers of plasma coating	59.3 ± 3.3
5 layers of plasma coating	54.7 ± 1.6

**Figure 10:**
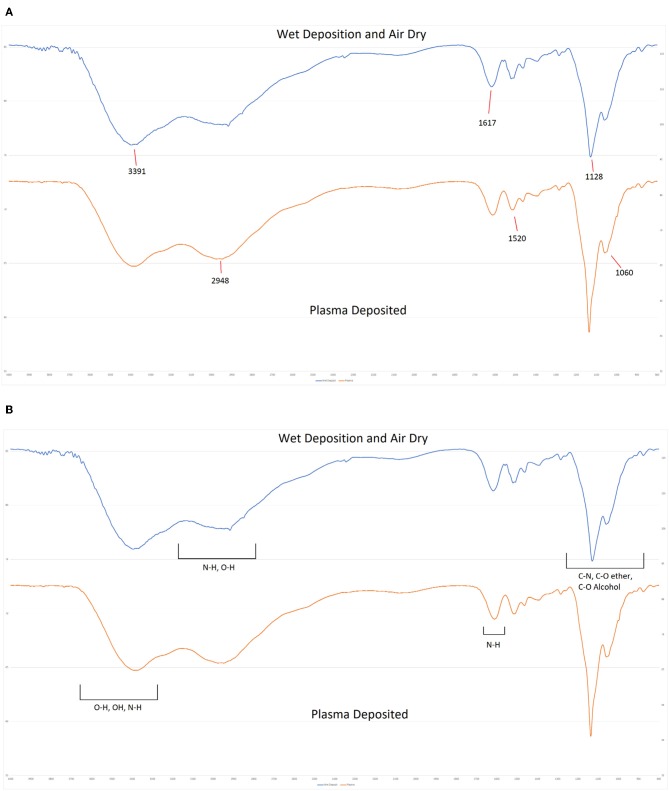
FTIR Spectra of plasma deposited gentamicin coating **(Top)** and air dried gentamicin solution **(Lower)**.

FTIR spectroscopy was also used to probe the chemistry of the deposited materials. Coatings were deposited directly onto sodium chloride discs using equivalent spray parameters. One coating was cured by powering on the plasma during deposition and the second coating was cured by allowing the wet solution to dry over a period of several hours. As shown in Figure 10, both deposits produced equivalent spectra. Both spectra are dominated by the overlapping C-O stretches from the multiple alcohol and ether groups between 1,000 and 1,080 cm^−1^ and a broad band from 3,000 to 3,600 cm^−1^ that can be attributed to a combination of N-H stretches of the amine groups and the O-H stretches from hydroxyl features. Additional features include the C-H derived peak centered at 2,981 cm^−1^ and the N-H bends at 1,615 and 1,515 cm^−1^ in both spectra. a weak C-N stretch from the amine groups can also be detected at 1,280 cm^−1^. No new features are evident in the spectrum of the plasma treated sample and there are no detectable peak shifts or alterations in intensity when compared to the air dried sample. This indicates that the plasma deposition process has not altered the chemistry of the deposited antibiotic.

## Discussion

Despite aseptic precautions and antibiotic prophylaxis during implantations, medical device-associated microbial infections still remain a major public health concern and may have serious consequences for the individual patient, including removal of the implant and significant economic costs (Xu et al., 2017). As systemic antibiotic treatment can be toxic to organ systems and promote the spread of antibiotic resistance, localized antimicrobial drug delivery is the subject of recent efforts to combat biofilm formation and infection for both preventive and treatment strategies (Jennings et al., 2015). The advantages of localized antibiotic delivery at a specific site enables administration of high local doses without exceeding the systemic toxicity level of the drug and risking renal and liver complications (Vasilev et al., 2011).

Antimicrobial coating of medical devices has emerged as a potentially effective method to mitigate device-related infections (Darouiche et al., 2007). Nikiforov et al. (2016) advise that to obtain an active antimicrobial activity, an antibiotic needs to be incorporated in the material surface. One novel approach for engineering a new class of biomaterials is using atmospheric pressure plasma processes for deposition of antimicrobial compounds. In this study we demonstrated that plasma deposition process can be used for a direct deposition of pharmaceutical drugs on to a range of surfaces without loss of functionality. To demonstrate this capability, two selected antibiotics—ampicillin and gentamicin, were deposited onto two types of surfaces—polystyrene microtiter plates and stainless steel coupons. Through control experiments, it was established that there was no separate antimicrobial or growth promoting effect of the plasma deposition process, where there was no antibiotic in the delivery stream. Therefore, plasma deposition process did not enhance the antibiotic efficacy as studied, apart from the flexibility offered through deposition on material surfaces. Trentin et al. (2014) investigated the effect of N_2_/H_2_ plasma surface modifications of polystyrene surface on growth and adhesion of a range of multidrug resistant bacteria. It was found that plasma treatments did not affect bacterial growth on solid surfaces for any of the tested strains based on optical density measurements, however, the exposure of polystyrene surfaces to plasma had an effect on bacterial adhesion—the treatment inhibited bacterial adhesion of strains that present hydrophilic surface (hydrophobicity index below 70%), reaching up to 83% adhesion inhibition for *K. pneumoniae*, 77% for *S. marcescens*, 65% for *S. aureus* and 48% for *E. cloacae*, during the first 24 h of incubation. Oppositely, *S. epidermidis*, which present hydrophobic surfaces (hydrophobicity index above 70%), retained its ability to adhere on the treated surfaces.

This study demonstrated that plasma deposition process retained the antibiotic activity. The antibiotics deposited on the test surfaces of microtiter plates retained good efficacy against planktonic cells and prevented biofilm formation of attached cells for up to 96 h (Figures 4, 5). Because of the difficulty in quantifying the exact amount of antibiotic deposited onto wells of a microtiter plate as a result of the deposition process, we compared residual efficacy to that of a calibration curve to provide an indication of the concentration range achieved of the functional antibiotic deposited during the process. Further chemical analysis is necessary to determine this aspect in a fully quantitative manner, as well as confirm structural retention post plasma deposition process. However, initial spectroscopic analysis using FTIR suggests that there are no detectable alterations in the chemical composition of the deposited materials. This is in keeping with studies of other biologics (Lackmann et al., 2018; Malinowski et al., 2018) that show that short term exposures to low levels of cold helium plasma as with the current deposition process investigated, had no detectable effect on chemistry and structure.

Similarly, plasma-deposited gentamicin on steel coupons was effective at reducing the populations of planktonic cells of bacteria when eluted in the surrounding liquid environment of both PBS and MHB (Figures 6, 7). The different types of medium could influence the elution of gentamicin from the coupons into the surrounding medium and/or affect the stability of the antibiotic in the solution. Since mechanism of action of gentamicin includes binding to the ribosomal subunit and protein synthesis inhibition (Tangy, 1985), this could explain why bacteria in PBS (not growing) are less affected - less protein synthesis is required in this “stationary” state than in actively dividing cells in MHB. Our preliminary tests showed no biofilm formation on the coupons; due to a very high antimicrobial activity of deposited gentamicin – after 24 h of incubation time cells of both strains were undetectable and biofilms could not be formed on gentamicin-coated coupons. Therefore, in this study the effect of antibiotic-coated surfaces on bacterial biofilm formation was tested only using microtiter plates. According to Vasilev et al. (2011), a release coating should provide fast initial release in the first 6 h after surgery to protect the site, while the immune system is weakened, followed by continuous “prophylactic” slow release. In our experiments it was observed that submerging gentamicin-coated steel coupons in either PBS or MHB liquids had an immediate antimicrobial effect and complete inactivation was achieved for both *E. coli* and *P. aeruginosa* within 24 h. Such rapid release of an entire or significant amount of antibiotics from the tested surfaces when in contact with liquids is not desirable as it can lead to negative side effects, especially the increased risk of drug-induced toxicity. Therefore, for the release in a controlled manner and prolonged efficacy of antibiotics, they should be deposited inside the matrix, which should be taken into consideration for future studies.

The elution of the antibiotic from the surface of gentamicin-coated steel coupons into PBS and MHB and antimicrobial activity of the solutions against both planktonic cells and bacterial biofilms was also investigated. Antimicrobial efficacy of MHB-gentamicin solutions was higher than PBS-gentamicin solutions—media formulation of MHB did not seem to impact on the antibiotic stability or provide conditions for microbial recovery and growth for up to 2 weeks duration. However, PBS is a more representative medium for potential future applications concerning antibiotic deposition to medical device and or implants. This proof of concept study was performed to determine if the approach used was feasible and efficient, therefore we employed a commonly used buffer solution (PBS) in comparison to medium recommended for antibiotic susceptibility testing by CLSI (MHB). The impact of more complex media containing serum should be examined to probe the degradation and chemical changes of antibiotics in a medium reflecting more realistic and challenging clinical conditions.

The surface of steel coupons subjected to plasma deposition of antibiotics increased in hydrophilicity; observed through a decrease of WCA by ~20% (Table 4). It is probable that an increase of hydrophilic properties can be used to achieve a better biomolecule adsorption on the surface and enhanced interactions with aqueous environment, enabling the active compound to be fully released to the surrounding solution. Plasma treatment has been previously reported to modify both stainless steel (Kim et al., 2003) and polystyrene surfaces (Mitchell et al., 2004) to be more hydrophilic.

In the future experiments the approach described in this study needs to be tested against Gram-positive bacteria, especially *Staphylococcus aureus*, frequently associated with medical-device related infections. For the present study, Gram-negative bacteria were selected, as generally they are more resistant to antibiotic treatment than Gram-positive. Selected *E. coli* and *P. aeruginosa* have higher MIC quality control ranges of gentamicin (0.25–1 and 0.5–2, respectively) as compared to *Staphylococcus aureus* (0.12–1) (CLSI, 2015). Although the results of this study demonstrate the retained efficacy against planktonic cells and prevented biofilm formation of attached cells on the test surfaces after plasma deposition of antibiotics, further work is necessary to determine if this approach can reduce the rate of implant-associated infections for both *in vivo* and in clinical applications.

## Conclusion

Overall, a novel plasma process for direct deposition of active pharmaceutics showed retention of antimicrobial effect and efficacy of the antibiotic-coated surfaces against *E. coli* and *P. aeruginosa*. Direct plasma deposition of antimicrobial compounds shows potential to functionalize a surface to prevent or control device associated infection. This presents an alternative to systemic antibiotic delivery that can be localized to a surgical or implant site and employs only the active antibiotic delivery stream. No independent antimicrobial or microbial growth promoting effects of the plasma deposition process were observed.

## Data Availability Statement

All datasets generated for this study are included in the article/supplementary material.

## Author Contributions

PB, AL, DB, LO'N, LH, and DZ conceived and designed the experiments. AL and DO'S performed the experiments. AL, DO'S, PB, and LO'N wrote the paper.

### Conflict of Interest

LO'N is affiliated with TheraDep Ltd. DO'S is affiliated with TheraDep Ltd and TU Dublin as an employment-based Ph.D. candidate. The remaining authors declare that the research was conducted in the absence of any commercial or financial relationships that could be construed as a potential.
